# Contributions to the knowledge of Chinese flea beetle fauna (II): *Baoshanaltica* new genus and *Sinosphaera* new genus (Coleoptera, Chrysomelidae, Galerucinae, Alticini)

**DOI:** 10.3897/zookeys.720.12715

**Published:** 2017-12-11

**Authors:** Yongying Ruan, Alexander S. Konstantinov, K. D. Prathapan, Xingke Yang

**Affiliations:** 1 Key Laboratory of Zoological Systematics and Evolution, Institute of Zoology, Chinese Academy of Sciences, Beijing 100101, China; 2 Postdoctoral Innovation Practice Base (College of Applied Chemistry and Biological Technology), Shenzhen Polytechnic, Shenzhen, China; 3 Systematic Entomology Laboratory, USDA, ARS, Washington DC, USA; 4 Department of Entomology, Kerala Agricultural University, Vellayani P.O., Trivandrum -695 522, Kerala, India

**Keywords:** Alticini, beetle diversity, China, flea beetles, moss-inhabiting, new genera, new species

## Abstract

Two new genera: *Baoshanaltica* **gen. n.** and *Sinosphaera* **gen. n**., and two new species: *Baoshanaltica minuta* **sp. n.** and *Sinosphaera aptera* **sp. n.** from south-west China are described and illustrated. *Baoshanaltica* is compared to the allied moss-inhabiting genera *Cangshanaltica* Konstantinov et al. and *Phaelota* Jacoby, in addition to *Minota* Kutschera. *Sinosphaera* is compared to *Sphaeroderma* Stephens, *Omeisphaera* Chen & Zia, *Jacobyana* Maulik, and *Kamala* Maulik.

## Introduction

As currently understood, Chinese flea beetle fauna consists of 102 genera and 856 species (Ruan and Yang 2015, Ruan et al. 2017). However, new taxa are still being discovered, particularly in mountainous south-west China (e.g. Yunnan and Sichuan) and in particular in the habitats that are poorly sampled for leaf beetles not just in China but throughout the world: leaf litter and moss cushions. So far 30 species from 15 genera are known to occur in moss cushions in the world (Damaška and Konstantinov 2016). A discovery of a new genus and species of flea beetles in Baoshan mountains in Yunnan brings the number to 31 and 16 respectively (Table 1). *Baoshanaltica minuta* sp. n. and *Sinosphaera aptera* sp. n. described in this paper bring the total number of flea beetle genera known to occur in China to 104 and species to 858.

**Table 1.** Flea beetle genera with species known to occur in moss cushions.

Genus name	Type species	Total number of species	Number of moss-inhabiting species	Biogeographic region
*Baoshanaltica* Konstantinov & Ruan, gen. n.	*Baoshanaltica minuta* Konstantinov & Ruan, sp. n.	1	1	Oriental
*Benedictus* Scherer, 1969	*Benedictus elisabethae* Scherer, 1969	27	7	Oriental
*Borinken* Konstantinov & Konstantinova, 2011	*Borinken elyunque* Konstantinov & Konstantinova, 2011	1	1	Neotropical
*Cangshanaltica* Konstantinov et al., 2013	*Cangshanaltica nigra* Konstantinov et al., 2013	2	2	Oriental
*Clavicornaltica* Scherer, 1974	*Clavicornaltica besucheti* Scherer, 1974	20	1	Oriental
*Ivalia* Jacoby, 1887	*Ivalia viridipennis* Jacoby, 1887	57	1	Oriental
*Kiskeya* Konstantinov & Chamorro-Lacayo, 2006	*Kiskeya baorucae* Konstantinov & Chamorro-Lacayo, 2006	3	3	Neotropical
*Minota* Kutschera, 1859	*Haltica obesa* Waltl, 1839	5	1	Palearctic
*Mniophila* Stephens, 1831	*Haltica muscorum* Koch, 1803	6	3	Palearctic
*Monotalla* Bechyné,1956	*Monotalla guadeloupensis* Bechyné, 1956	6	2	Neotropical
*Nicaltica* Konstantinov et al., 2009	*Nicaltica selvanegra* Konstantinov et al., 2009	1	1	Neotropical
*Paraminota* Scherer, 1989	*Paraminota minima* Scherer, 1989	2	1	Oriental
*Paraminotella* Döberl & Konstantinov, 2003	*Paraminota nepalensis* Döberl, 1991	2	1	Oriental
*Phaelota* Jacoby, 1887	*Phaelota semifasciata* Jacoby, 1887	16	3	Oriental
*Stevenaltica* Konstantinov et al., 2014	*Stevenaltica normi* Konstantinov et al., 2014	2	2	Neotropical
*Ulrica* Scherer, 1962	*Sparnus minutus* Jacoby, 1889	5	1	Neotropical

*Baoshanaltica* resembles another moss-inhabiting genus *Cangshanaltica* Konstantinov et al., 2013 and is compared with it, besides *Minota* Kutschera and *Phaelota* Jacoby. *Sinosphaera* gen. n. is allied to *Jacobyana* Maulik, *Kamala* Maulik, *Omeisphaera* Chen & Zia, and *Sphaeroderma* Stephens. Morphological comparisons between the new genera and their allies are given in “Results” part of the paper.

## Materials and methods

Observations of the male genitalia and habitus were made with a Zeiss Discovery V20 microscope and digital images were taken with an AxioCam HRC digital camera attached to it. Female genitalia were dissected and mounted on slides in glycerin, and photographed with Leitz Diaplan Microscope and the camera module of Blackberry Q10 mobile phone (with a resolution of 800MP). The morphological terminology follow Konstantinov (1998).

Abbreviations of collections:

**IZCAS** Institute of Zoology, Chinese Academy of Sciences, Beijing, China.

**USNM** National Museum of Natural History, Washington DC, USA.

## Results

### Baoshanaltica

Taxon classificationAnimaliaColeopteraChrysomelidae

Konstantinov & Ruan gen. n.

http://zoobank.org/FE1DABB6-4EA4-4652-A213-B620A9390AEC

Figs 1 , 2 , 3

#### Type species.

Baoshanaltica minuta Konstantinov & Ruan, sp. n.

#### Etymology.

We name this genus after its type locality: Baoshan (保山) mountains, Yunnan province. The name is feminine.

#### Distribution.

China.

#### Host plant.

Possibly unknown species of moss.

**Figure 1.**
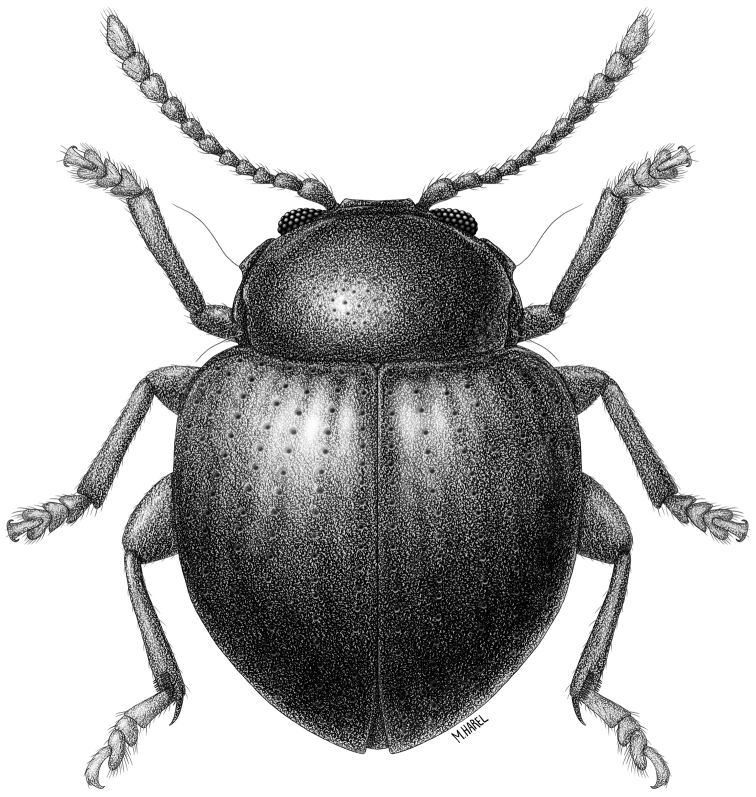
*Baoshanaltica minuta* sp. n. (habitus).

#### Description.

Body color and proportions. Body unicolorous, brown to dark brown, without slight metallic luster. Body ovate in dorsal view, highly convex in lateral view. Body length 1.40–1.55 mm (n=2). Body width (widest point of elytra) 1.05–1.10 mm. Body length to width, ratio 1.35–1.45. Pronotum width to length, ratio 1.50–1.60. Pronotum width at base to width at apex, ratio 1.05–1.10. Elytron length (measured along suture) to width of both, ratio 0.90–1.00. Elytron and abdomen length to height of the body (in lateral view), ratio 1.30–1.40. Length of elytron to length of pronotum, ratio 2.75–2.85. Width of elytra at base (measured across middle of humeral calli) to width of pronotum at base, ratio 1.25–1.30.

Head. Surface glabrous, dark brown, shiny, without punctures, except for supraorbital. Antennal calli poorly delimited with supracallinal, midfrontal, supraantennal, and suprafrontal sulci absent to poorly developed. Frontal ridge wider between antennal sockets than near clypeus. Each side of frontal ridge with few white, long setae. Top of frontal ridge separated from vertex by a more or less round impression. Width of frontal ridge to antennal sockets (counting surrounding ridges), ratio 2.50–2.60. Frontal ridge in lateral view moderately convex. Frontal ridge and anterofrontal ridge in frontal view form nearly straight angle. Vertex obviously concave at its lower part near frontal ridge.

Orbit as wide as transverse diameter of antennal socket. Inner margin of eye straight. Distance between eyes (just above antennal sockets) to transverse diameter of eye in frontal view, ratio 3.50–3.60. Longitudinal diameter of eye to transverse diameter of eye in frontal view, ratio 1.95–2.05. Sides of head below eyes converging ventrally. Labrum flat with 2 pairs of setae, without projections in middle. Anterior margin of labrum with shallow emargination in middle. Apical maxillary palpomere conical. Supraorbital pore well developed. Clypeus band-like in shape. Antennal sockets situated about middle of eye. Distance between antennal sockets to transverse diameter of one antennal socket, ratio 2.50–2.60.

Antennae stout and short, only slightly stretch over pronotum. Number of antennomeres: 11. First antennomere slightly as long as or slightly shorter than next two combined. Antennomere 2 longer than 3. Antennomere 5 about as long as antennomeres 4 and 6 separately. Distal antennomeres robust, wider than middle ones. Antennomere 7 in males with lobe projecting dorsally. Length to width of antennomere 9, ratio 1.20–1.25. Length to width of antennomere 10, ratio 1.05–1.10. Length to width of antennomere 11, ratio 1.45–1.55.

Prothorax. Pronotal surface glabrous, with a shallow and poorly defined transverse impression and two poorly defined lateral impressions near base. Pronotal punctures as large as elytral ones, their diameter 2–3 times smaller than distance between them. Anterolateral callosity of pronotum well developed, long, facing anterolaterally with obtuse denticle posteriorly. Anterior setiferous pore of pronotum situated close to middle of lateral margin. Sides of pronotum curved, somewhat sinuate. Pronotal base straight. Lateral margin of pronotum complete and strongly explanate. Posterolateral setiferous pore of pronotum protruding laterally beyond lateral margin.

Procoxal cavities open. Lateral sides of intercoxal prosternal process concave in middle, apex slightly wider than middle. Posterior end of intercoxal prosternal process slightly convex. Intercoxal prosternal process slightly extends beyond procoxae. Intercoxal prosternal process normally wide. Width of intercoxal prosternal process between procoxae to length of procoxa, ratio 0.65–0.70.

Elytra. Humeral calli absent. Hind wings absent. Impressions or ridges on elytron absent. Elytron with small punctures arranged in 8 rows; scutellar row of punctures absent. Interspaces slightly costate. Scutellum present, extremely small, triangular. Elytron with apex acute, covering entire abdomen. Sides strongly and evenly convex. Epipleura oblique outwardly, gradually narrowing from base to apex, nearly reaching apex. Width of epipleura greater than that of profemur. Epipleura basally much wider than apically. Elytra at base wider than base of pronotum.

Venter. Meso- and metasterna more or less flat, without elevated projection in middle. Metasternum slightly projecting forward. Abdominal ventrites 1 and 2 not fused. Abdominal ventrite 1 as long as remaining ventrites together. Abdominal ventrite 5 longer than ventrites 4, 3 and 2 together. First abdominal ventrite between coxa without longitudinal ridges. Anterior end of first abdominal ventrite normally wide and truncate.

Legs. Apical spur of protibia and mesotibia absent. Apical spur of metatibia relatively short and slender. Metafemoral spring present. Claw simple. Apical part of hind and middle tibia without excavation. Length (not counting trochanter) to maximum width of metafemur, ratio 1.90–1.95. Length to width of metatibia in lateral view, ratio 7.70–7.80. Width of metatibia at base to width at apex in dorsal view, ratio 0.50–0.60. Length of metatibia to length of first metatarsomere, ratio 4.50–4.60. Metatibia generally straight. Metatibia in cross section around its middle more or less cylindrical. Dorsal side of metatibia without sharp edge or small denticles. Metatarsomere 1 attached to apex of metatibia. Length of metafemur to metatibia, ratio 1.25–1.35. First protarsomere of male, length to width, ratio (in dorsal view) 1.40–1.50. Length of first protarsomere to length of second protarsomere, ratio 1.80–1.90. Width of first protarsomere to width of second protarsomere, ratio 0.95–1.00. Tarsomere 3 incised, deeply bilobed and slightly elongate. First metatarsomere of male, length to width, ratio (in dorsal view) 2.20–2.30. Length of first metatarsomere much less than half of metatibial length. First and rest three metatarsomeres make more or less straight line. Length of first metatarsomere to length of second metatarsomere, ratio 2.90–3.00. Width of first metatarsomere to width of second metatarsomere, ratio 1.00–1.05. Length of fourth metatarsomere to length of third metatarsomere, ratio 1.40–1.45.

Genitalia. Aedeagus slender, flattened in cross section, strongly and evenly curved in lateral view. Apex abruptly narrowed.

**Figure 2.**
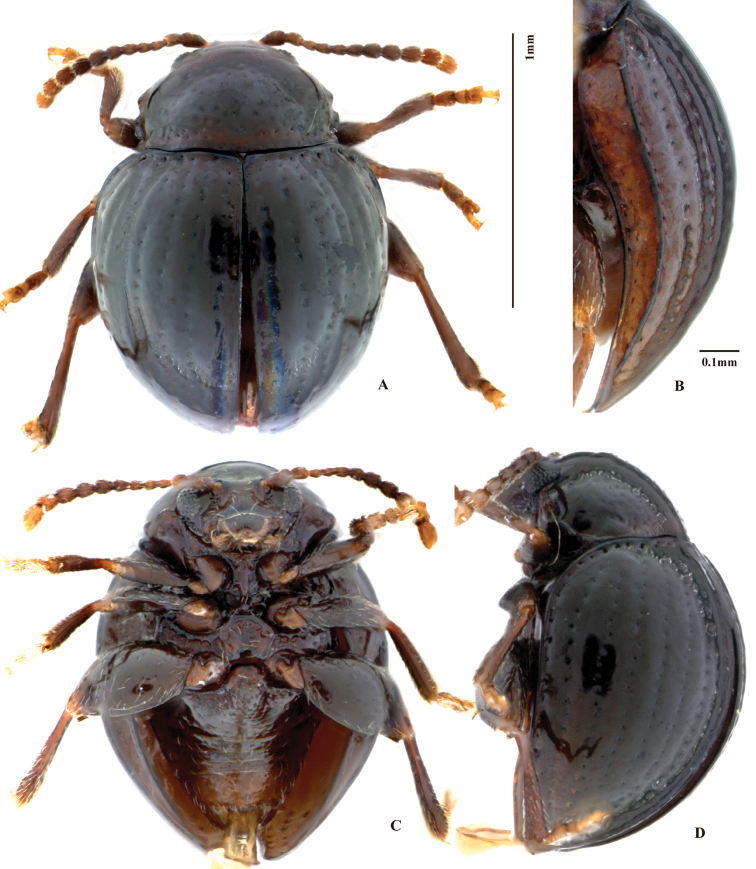
*Baoshanaltica minuta* sp. n. **A** habitus (holotype, male) **B** epipleuron **C** venter **D** lateral view of body.

#### Remarks.

In the general shape, *Baoshanaltica* resembles moss-inhabiting flea beetles from the genus *Cangshanaltica* Konstantinov et al., 2013, discovered in a neighboring mountain ridge in Yunnan and later found in northern Thailand (Damaška and Konstantinov 2016). However, *Baoshanaltica* can be differentiated from the latter by the following characters: frontal ridge generally narrow, wider between antennal sockets than near clypeus, forming nearly straight angle with anterofrontal ridge (in *Cangshanaltica* frontal ridge wide, as wide between antennal sockets as near clypeus, forming one solid structure with anterofrontal ridge); anterolateral callosity of pronotum straight, facing anterolaterally with obtuse denticle posteriorly (in *Cangshanaltica* callosity convex, facing more anteriorly than laterally without denticle posteriorly); elytra with regular rows of punctures and convex interspaces (in *Cangshanaltica* elytral punctures placed irregularly and elytra without convex interspaces); metatibial spur short and slender (in *Cangshanaltica* metatibial spur long and more robust). From *Minota*, with which *Baoshanaltica* shares similarly shaped frontal ridge, absence of humeral calli, regular elytral punctuation and wide epipleuron, it can be differentiated by: presence of anterofrontal ridge (absent in *Minota*); poorly developed supracallinal sulci (well developed in *Minota*); and open procoxal cavities (closed in *Minota*). *Baoshanaltica* resembles apterous species of *Phaelota*, which are also moss-inhabiting. Both share characters such as two pairs of labral setae, robust distal antennomeres, presence of antebasal transverse impression on pronotum, regular elytral punctation, wide elytral epipleura reaching almost up to elytral apex etc. However, *Baoshanaltica* can be easily differentiated from *Phaelota* based on the modified 7^th^ antennomere in males (unmodified in *Phaelota*), open procoxal cavities (closed in *Phaelota*), metatibia which is not sexually dimorphic (sexually dimorphic in *Phaelota*) and eyes separated by a distance of 3.50–3.60 times transverse diameter of one eye (eyes separated by a distance of 1.6–2.3 times transverse diameter of one eye in *Phaelota*).

**Figure 3.**
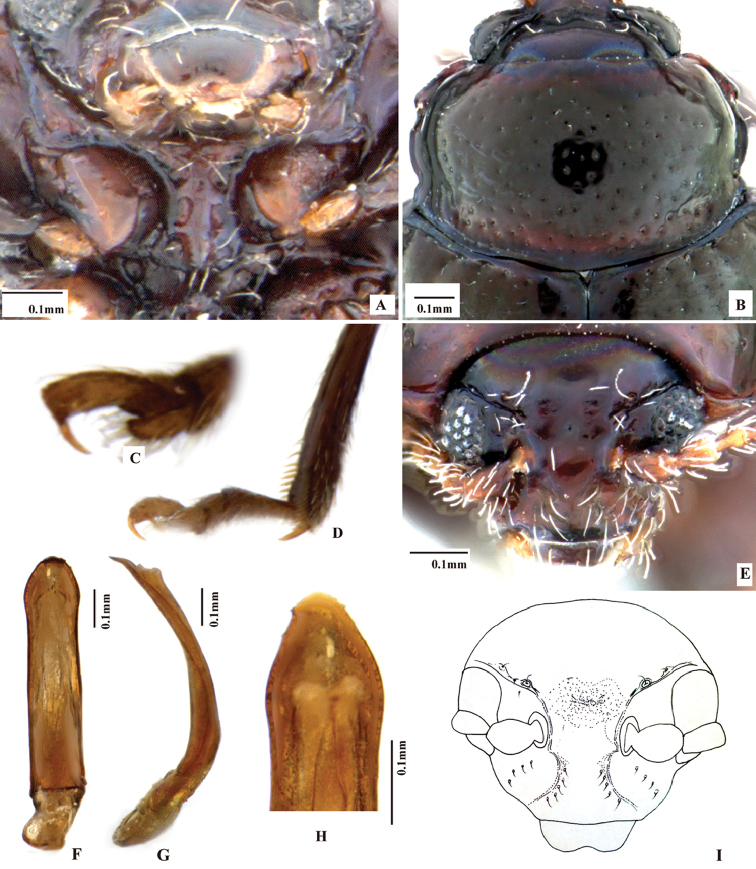
*Baoshanaltica minuta* sp. n. **A** prosternum **B** pronotum **C** claw **D** tarsi of hind leg **E** frontal view of head **F** Aedeagus, ventral view **G** aedeagus, lateral view **H** apical part of aedeagus, dorsal view **I** frontal view of head (drawing).

### Baoshanaltica minuta

Taxon classificationAnimaliaColeopteraChrysomelidae

Konstantinov & Ruan sp. n.

http://zoobank.org/4CC8CD22-1BFE-4224-AD84-CC5B395B6D5B

Figs 1 , 2 , 3

#### Etymology.

The new species is named after its small body size.

#### Distribution.

China.

#### Host plant.

Possibly unknown species of moss.

#### Type material.

**Holotype**, ♂ (IZCAS), labels: 1) Yunnan, 60 km W Baoshan, Lujian Zhen, 11.VI.2012, N24.55'736 E99.48'332, h-2383 m, moss sifted, leg. A. Konstantinov. 2) Holotype. 3) *Baoshanaltica minuta* des. Konstantinov & Ruan, 2016.

#### Paratype.

1♂ (USNM), labels: 1) Yunnan, 60 km W Baoshan, Lujian Zhen, 111.VI.2012, N24.55'736 E99.48'332, h-2383 m, moss sifted, leg. A. Konstantinov. 2) Paratype *Baoshanaltica minuta* des. Konstantinov & Ruan, 2016.

#### Description.

Dorsum and venter uniformly brown to dark brown, without metallic luster. Body oval in dorsal view, highly convex in lateral view. Body length: 1.40–1.55 mm (n=2). Body width (widest point of elytra): 1.05–1.10 mm.

Antennal calli poorly delimited with supracallinal, midfrontal, supraantennal, and suprafrontal sulci absent to poorly developed. Frontal ridge wider between antennal sockets than near clypeus. Top of frontal ridge separated from vertex by more or less round impression. Width of frontal ridge to antennal sockets (counting surrounding ridges), ratio 2.50–2.60. Vertex shiny, without punctures, except for supraorbital, obviously concave at its lower part near frontal ridge. Distance between eyes (just above antennal sockets) to transverse diameter of eye in frontal view, ratio 3.50–3.60. Longitudinal diameter of eye to transverse diameter of eye in frontal view, ratio 1.95–2.05. Sides of head below eyes converging ventrally. Distance between antennal sockets to transverse diameter of one antennal socket, ratio 2.50–2.60.Proportions of *antennomeres* as follows: 13:7:6:7:7:6:8:8:9:10:15. Length to width of antennomere 9, ratio 1.20–1.25. Length to width of antennomere 10, ratio 1.05–1.10. Length to width of antennomere 11, ratio 1.45–1.55.

Pronotal surface glabrous, with punctures as large as elytral ones, their diameter 2–3 times smaller than distance between them. Sides of pronotum curved, somewhat angulate near middle. Posterolateral setiferous pore of pronotum protruding laterally beyond lateral margin. Elytral humeral callus absent. Hind wings absent. Elytron with small punctures arranged in 8 rows, scutellar row absent. Interspaces slightly costate.

Length (not counting trochanter) to maximum width of metafemur, ratio 1.90–1.95. Length to width of metatibia in lateral view, ratio 7.70–7.80. Width of metatibia at base to width at apex in dorsal view, ratio 0.50–0.60. Length of metatibia to length of first metatarsomere, ratio 4.50–4.60. First protarsomere of male, length to width, ratio (in dorsal view), ratio 1.40–1.50. First metatarsomere of male, length to width, ratio (in dorsal view), ratio 2.20–2.30. Length of first metatarsomere to length of second metatarsomere, ratio 2.90–3.00.

Aedeagus slender, flattened in cross section, with long membranous window ventrally. In lateral view aedeagus strongly and evenly curved with apex abruptly bent ventrally. Apical denticle absent.

### Sinosphaera

Taxon classificationAnimaliaColeopteraChrysomelidae

Konstantinov & Ruan gen. n.

http://zoobank.org/2E610C26-7999-4301-85F2-7E67C2E41793

Figs 4 , 5 , 6

#### Type species.

*Sinosphaera aptera* Konstantinov & Ruan, new species.

#### Etymology.

The new genus is named for its spherical body outline. The name is feminine.

#### Distribution.

China.

#### Host plant.

Unknown.

**Figure 4.**
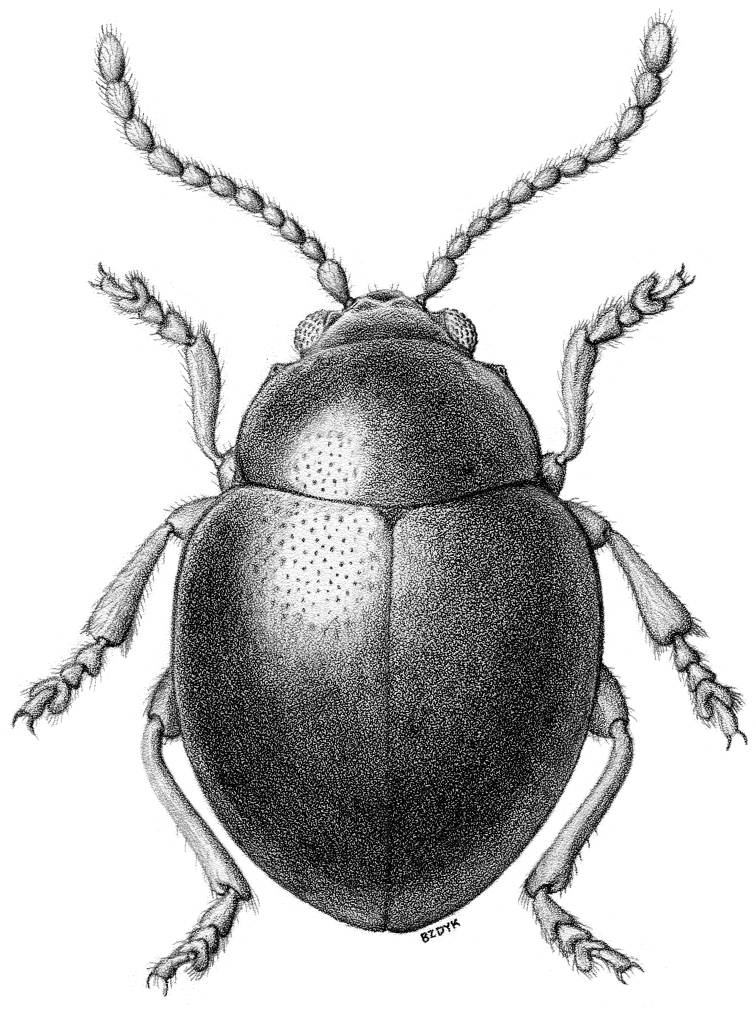
*Sinosphaera aptera* sp. n. (habitus).

#### Description.

Body color and proportions. Dorsal surface without hair, glabrous, metallic bluish or greenish, shiny, pronotum very slightly darker than elytra. Ventral surface dark brown. Body spherical in dorsal view, moderately and evenly convex in lateral view. Body length: 2.60–2.80 mm (n=2). Body width (widest point of elytra): 1.80–2.00 mm. Body length to width, ratio 1.35–1.45. Pronotum width to length, ratio 1.90–2.00. Pronotum width at base to width at apex, ratio 1.25–1.35. Elytron length (measured along suture) to width of both, ratio 0.95–1.05. Elytron and abdomen length to height of body (in lateral view), ratio 1.50–1.55. Length of elytron to length of pronotum, ratio 2.60–2.70. Width of elytra at base (measured across middle of humeral calli) to width of pronotum at base, ratio 1.05–1.10.

Head. Vertex metallic blue, shiny, with extremely minute and distantly placed punctures. Vertex with small indentation or transverse impression above frontal ridge. Frontal ridge short, as wide between antennal sockets as near anterofrontal ridge, entering between antennal calli. Top of frontal ridge meet and merge with vertex. Width of frontal ridge to antennal sockets (counting surrounding ridges), ratio 2.30–2.35. Frontal ridge in lateral view moderately convex. Frontal ridge and anterofrontal ridge in frontal view gradually merge into each other. Frontal ridge and vertex in lateral view form convex line. Anterofrontal ridge lower near frontal ridge, higher laterally, concave in middle.

Antennal calli more or less triangular, at same level as surface of vertex, separated from each other by tip of frontal ridge, entering interantennal space. Width to length of antennal callus, ratio 1.40–1.45. Length of antennal calli smaller than length of frontal ridge. Supracallinal sulcus slightly and evenly curved, poorly developed. Suprafrontal sulcus straight, poorly developed. Supraorbital and orbital sulci very short and deep. Supraantennal sulcus long, stronger than supracallinal, but not as deep as orbital sulcus. Frontolateral sulcus poorly developed.

Orbit normally wide, as wide as transverse diameter of antennal socket. Supraorbital pore poorly developed, unrecognized. Inner margin of eye straight to very slightly concave in middle. Distance between eyes (just above antennal sockets) to transverse diameter of eye in frontal view, ratio 4.20–4.30. Longitudinal diameter of eye to transverse diameter of eye in lateral view, ratio 2.00–2.10. Sides of head below eyes converging ventrally. Labrum flat, without projections in middle. Anterior margin of labrum entire and straight. Number of labral setae: 3 pairs. Apical maxillary palpomere conical, length to width, ratio 1.70–1.80. Preapical maxillary palpomere proximally narrower than distally. Length to width, of preapical maxillary palpomere, ratio 1.00–1.05. Length of apical to preapical maxillary palpomeres, ratio 1.55–1.60. Clypeus band like. Antennal sockets situated nearly at middle level of eye. Distance between antennal sockets to transverse diameter of one antennal socket, ratio 2.70–2.80.

Antennae. Antennae filiform, stretching over pronotum but not reaching half of elytron. Number of antennomeres 11. Length of antennomere 1 slightly greater than next two antennomeres combined. Antennomere 2 robust, longer and wider than 3. Antennomere 5 slightly longer than antennomere 4 and slightly shorter than 6 separately. Distal antennomeres slightly wider than middle ones. Length to width of antennomere 9, ratio 1.40–1.45. Length to width of antennomere 10, ratio 1.15–1.20. Length to width of antennomere 11, ratio 2.40–2.45.

Prothorax. Pronotal surface glabrous, with 2 antebasal, barely visible longitudinal impressions, laterally. Anterolateral corners of pronotum projected prominently forward. Anterolateral callosity of pronotum strongly developed, ovoid, without angulation, facing anteriorly. Anterior setiferous pore of pronotum situated close to anterior margin. Sides of pronotum weakly convex, strongly converging anteriorly. Pronotal base evenly convex. Lateral margin of pronotum complete and narrowly explanate. Posterolateral setiferous pore of pronotum not protruding laterally beyond lateral margin.

Procoxal cavities closed. Lateral sides of intercoxal prosternal process concave in middle, with apex much wider than middle. Posterior end of intercoxal prosternal process nearly straight. Intercoxal prosternal process normally wide, convex along its length, without ridge, slightly extends beyond procoxae. Width of intercoxal prosternal process between procoxae to length of procoxa, ratio 0.70–0.75.

Elytra. Elytra without humeral calli. Hind wings absent. Elytra at base wider than base of pronotum. Impressions or ridges on elytron absent. Elytron with punctures minute and confused. Scutellum present, small. Elytron with apex rounded, covering entire abdomen. Elytra with strongly and evenly convex sides. Epipleura oblique outwardly. Epipleura gradually narrowing from base to apex. Width of epipleura about equal to that of profemur. Epipleura basally much wider than apically. Epipleura reaches end of elytron.

**Figure 5.**
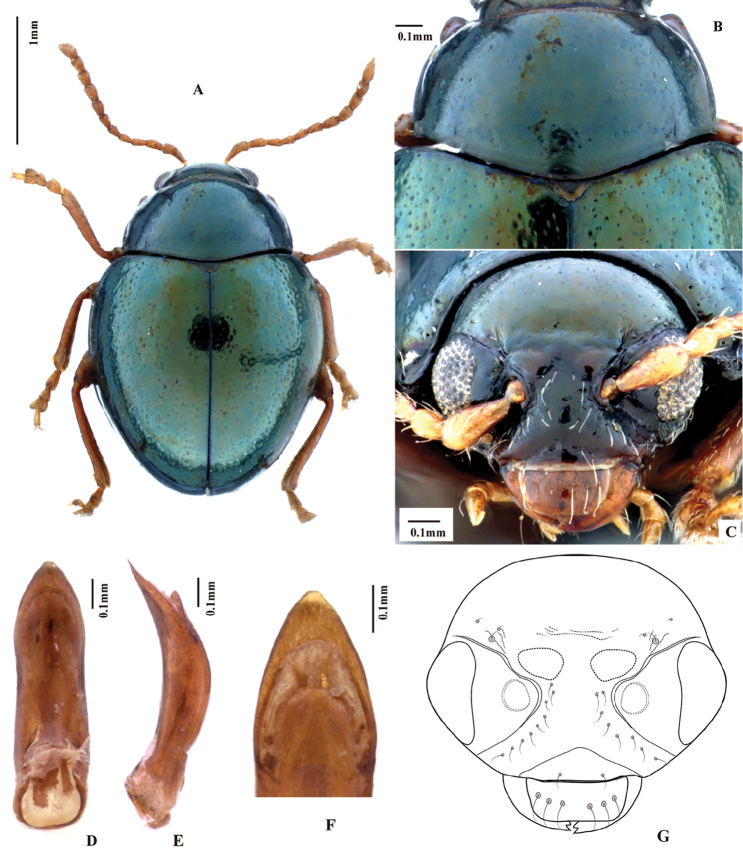
*Sinosphaera aptera* sp. n. **A** habitus (holotype, male) **B** prothorax, dorsal view **C** head, frontal view **D** aedeagus, ventral view **E** aedeagus, lateral view **F** apical part of aedeagus, dorsal view **G** frontal view of head (drawing).

Venter. Metasternum projecting forward, covers and conceals mesosternum. Metasternum without elevated projection in middle. Abdominal ventrites 1 and 2 not fused. Abdominal ventrite 1 longer than ventrites 2, 3, and 4 together. Abdominal ventrite 5 as long as ventrites 4 and 3 together. First abdominal ventrite between coxae without longitudinal ridges. Anterior end of first abdominal ventrite extremely wide and slightly convex.

Legs. Pro- and mesotibia without apical spur. Apical spur of metatibia tiny, slightly larger than other bristles. Metafemoral spring present. Claw appendiculate. Apical part of hind and middle tibia without excavation. Length (not counting trochanter) to maximum width of metafemur, ratio 2.50–2.55. Length to width of metatibia in lateral view, ratio 7.00–7.10. Width of metatibia at base to width at apex in dorsal view, ratio 0.60–0.65. Length of metatibia to length of first metatarsomere, ratio 4.65–4.70. Metatibia generally straight. Metatibia in cross section around its middle more or less cylindrical, flattens only very close to apex and also abruptly widens near apex in dorsal view. Dorsal side of metatibia without sharp edge or small denticles. Metatarsomere 1 attached to apex of metatibia. Length of metafemur to metatibia, ratio 1.10–1.20. First protarsomere of male, length to width, ratio (in dorsal view) 1.20–1.30. Length of first protarsomere to length of second protarsomere, ratio 1.70–1.75. Width of first protarsomere to width of second protarsomere, ratio 1.05–1.10. Incision of tarsomere 3 as long as wide. Tarsomere 3 subtriangular. First metatarsomere of male, length to width, ratio (in dorsal view) 1.90–2.00. Length of first metatarsomere much less than half of metatibial length. First and rest three metatarsomeres make more or less straight line. Length of first metatarsomere to length of second metatarsomere, ratio 1.50–1.55. Width of first metatarsomere to width of second metatarsomere, ratio 0.95–1.00. Length of fourth metatarsomere to length of third metatarsomere, ratio 0.95–1.00.

Genitalia. Aedeagus robust and short, slightly flattened in cross section, evenly and moderately curved, with apex gradually narrowed.

#### Remarks.

*Sinosphaera* resembles *Omeisphaera* Chen & Zia and *Sphaeroderma* Stephens in having ovate body shape and similar, forward facing, anterolateral callosities of the pronotum. *Sinosphaera* can be easily differentiated from these genera by the short antebasal, lateral impression on the pronotum and closed procoxal cavities. They are open in *Omeisphaera* and *Sphaeroderma*, which are also lacking antebasal, lateral impression on the pronotum. In *Sinosphaera*, antennal calli are separated from each other by the top of the frontal ridge. They are connected in *Omeisphaera* and *Sphaeroderma*. *Sinosphaera* resembles *Kamala* in having ovate body shape, absence of humeral calli and closed procoxal cavities. However, they can be easily separated by the lack of antebasal impressions on pronotum (short longitudinal antebasal impressions present in *Kamala*), confused elytral punctation (elytral punctures form rows in *Kamala*) and the distal antennomeres only slightly wider than the middle ones (distal antennomeres form a dilated club in *Kamala*). *Jacobyana* superficially resembles *Sinosphaera* in being rounded, convex and posteriorly narrowed. Minutely punctate vertex and frons (vertex and frons coarsely punctate in *Jacobyana*), evenly convex posterior margin of pronotum (posterior margin of pronotum bisinuate in *Jacobyana*), closed procoxal cavities (procoxal cavities open in *Jacobyana*), confused elytral punctation (elytral punctures regularly arranged in *Jacobyana*) and absence of hindwings and humeral calli (both present in *Jacobyana*) will separate these two genera.

**Figure 6.**
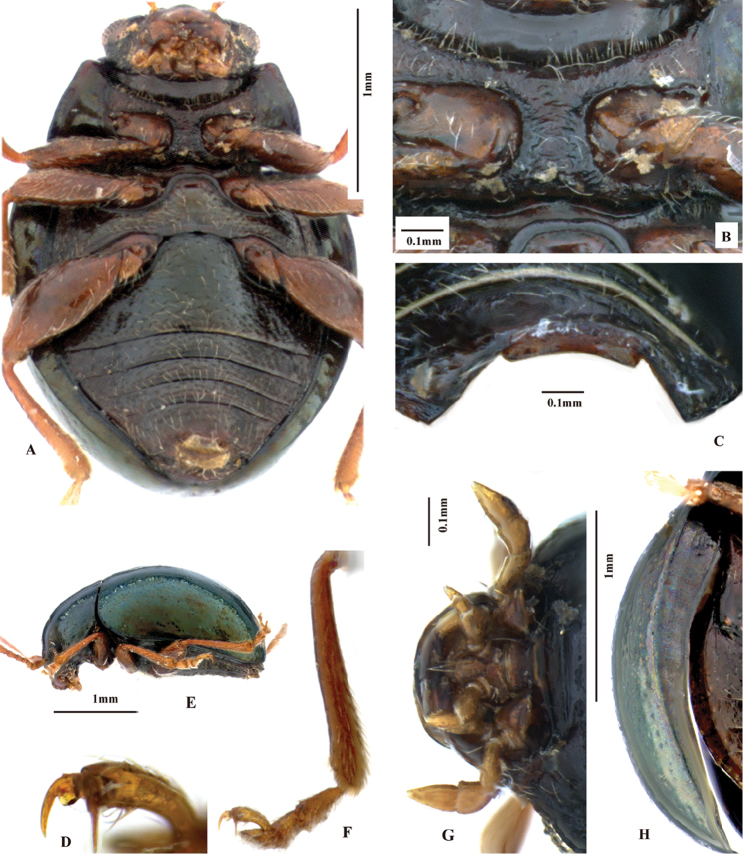
*Sinosphaera aptera* sp. n. **A** ventral view of holotype **B** prosternum **C** last ventrite of male **D** claw **E** lateral view of holotype **F** lateral view of hind tibia and tarsi **G** maxillary palpi and labial palpi **H** epipleuron.

### Sinosphaera aptera

Taxon classificationAnimaliaColeopteraChrysomelidae

Konstantinov & Ruan sp. n.

http://zoobank.org/85A7DF16-182B-44D1-A855-231DF85E5209

Figs 4 , 5 , 6

#### Etymology.

This species is named after the absence of hindwings.

#### Distribution.

China.

#### Host plant.

Unknown.

#### Type material.

**Holotype**, ♂ (IZCAS), labels: 1) CH, Sichuan, right bank of r. trib. of Lanhegou River, SSW of Jimi Vill., 2200–2600 m, 25.VI.2000, Belousov, Kabak, Davidian. 2) Holotype. 3) *Sinosphaera aptera* des. Konstantinov & Ruan, 2016.

#### Paratypes.

5♂ (USNM), labels: 1) CH, Sichuan, right bank of r. trib. of Lanhegou River, SSW of Jimi Vill., 2200–2600 m, 25.VI.2000, Belousov, Kabak, Davidian. 2) Paratype *Sinosphaera aptera* des. Konstantinov & Ruan, 2016.

#### Description.

Dorsal surface glabrous, metallic bluish or greenish, pronotum slightly darker than elytra. Body spherical in dorsal view, moderately and evenly convex in lateral view. Body length: 2.60–2.80 mm (n=2). Body width (widest point of elytra): 1.80–2.00 mm. Body length to width, ratio 1.35–1.45. Pronotum width to length, ratio 1.90–2.00. Pronotum width at base to width at apex, ratio 1.25–1.35. Elytron length (measured along suture) to width of both, ratio 0.95–1.05. Length of elytron to length of pronotum, ratio 2.60–2.70. Width of elytra at base (measured across middle of humeral calli) to width of pronotum at base, ratio 1.05–1.10.

Vertex shiny, with extremely minute and distantly placed punctures bearing small setae, punctures larger at base of vertex. Anterofrontal ridge with wrinkles on surface facing antennal calli.

Antennae with antennomeres of following proportions: 20:10:8:10:9:9:10:11:10: 10:15.

Distal antennomeres slightly wider than middle ones. Length to width of antennomere 9, ratio 1.40–1.45. Length to width of antennomere 10, ratio 1.15–1.20. Length to width of antennomere 11, ratio 2.40–2.45.

Pronotal surface glabrous with small, evenly spaced punctures, smaller than elytral punctures. Sides of pronotum weakly convex, strongly converging anteriorly. Pronotal base evenly convex.

Humeral calli of elytra absent. Hind wings absent. Elytral punctures larger than pronotal, confused.

Length (not counting trochanter) to maximum width of metafemur, ratio 2.50–2.55. Metatibia slender, its length to width in lateral view, ratio 7.00–7.10. Length of metatibia to length of first metatarsomere, ratio 4.65–4.70. First protarsomere of male, length to width, ratio (in dorsal view), 1.20–1.30. Length of first protarsomere to length of second protarsomere, ratio 1.70–1.75. Width of first protarsomere to width of second protarsomere, ratio 1.05–1.10.

Aedeagus relatively short with more or less convex ventral side and narrow groove stretching from basal opening to about 1/3 from apex. Apex gradually narrowed. Apical denticle of aedeagus absent.

## Supplementary Material

XML Treatment for Baoshanaltica

XML Treatment for Baoshanaltica minuta

XML Treatment for Sinosphaera

XML Treatment for Sinosphaera aptera
